# Impact of overweight on spatiotemporal gait parameters and lower-limb biomechanics in functional ankle instability

**DOI:** 10.3389/fphys.2026.1710727

**Published:** 2026-04-21

**Authors:** Dunbing Huang, Chen Zhang, Jiaqi Wang, Hongfei Ren, Zhenhua Wu, Lihong Li, Xiaohua Ke, Zhonghua Lin, Cai Jiang

**Affiliations:** 1 Encephalopathy and Rehabilitation Center, The Second Affiliated Hospital of Zhejiang Chinese Medical University, Hangzhou, Zhejiang, China; 2 Rehabilitation Medicine Department, The Third Affiliated Hospital of Zhejiang Chinese Medical University, Hangzhou, Zhejiang, China; 3 Department of Rehabilitation Medicine, Shanghai Fourth People’s Hospital, School of Medicine, Tongji University, Shanghai, China; 4 Rehabilitation Medicine Center, Fuzhou University Affiliated Provincial Hospital, Fuzhou, China; 5 Rehabilitation Medicine Center, Shengli Clinical Medical College of Fujian Medical University, Fuzhou, China

**Keywords:** biomechanics, functional ankle instability, gait analysis, gait variability, overweight

## Abstract

**Background:**

Ankle sprains are common in physically active populations and may progress to functional ankle instability, which can impair gait. Excess body mass increases lower-limb loading and may further compromise walking function in this population.

**Objective:**

To examine whether overweight status influences spatiotemporal gait parameters, gait deviation/variability indices, and sagittal-plane hip, knee, and ankle biomechanics during level walking in individuals with functional ankle instability.

**Methods:**

In this cross-sectional comparative analysis, forty-four adults with functional ankle instability were enrolled (22 normal-weight and 22 overweight). Three-dimensional motion analysis was used during self-selected speed walking to quantify spatiotemporal parameters, gait deviation/variability indices, and sagittal-plane hip, knee, and ankle kinematics and kinetics. Multiple comparisons across gait outcomes were controlled using a Holm-Bonferroni correction (family-wise error rate 0.05).

**Results:**

After Holm–Bonferroni correction, spatiotemporal parameters did not differ significantly between groups. For gait deviation/variability indices, several measures showed nominal (unadjusted) between-group differences, but none remained significant after correction. For discrete sagittal-plane biomechanics, the overweight group demonstrated lower peak hip flexion after correction (P_adj < 0.05), whereas hip flexion at toe-off showed a nominal difference but did not remain significant after correction (P_adj ≥ 0.05).

**Conclusion:**

After multiplicity control, overweight status was mainly associated with reduced hip flexion-related mechanics rather than consistent spatiotemporal or deviation/variability alterations in adults with functional ankle instability.

## 1 Introduction

Ankle injuries are among the most common musculoskeletal injuries in physically active individuals (Biz et al., 2022). Globally, ankle sprains occur at an estimated rate of approximately one per 10,000 people per day. Moreover, ankle sprains show a high recurrence rate (up to 73.5%), and lateral ankle sprains can lead to substantial time loss and persistent disability in as many as 60% of patients (Lee et al., 2022). Approximately 40% of individuals with lateral ankle sprains experience recurrent sprains and residual symptoms (e.g., pain and perceived instability) lasting at least 12 months (Ardakani et al., 2019). A frequent long-term consequence is chronic ankle instability (CAI). One prevalent subtype is functional ankle instability (FAI), characterized primarily by sensorimotor dysfunction, manifesting as recurrent “giving way,” pain, and functional limitations, despite ankle joint motion often remaining within a clinically normal range (Huang et al., 2021). Injury-related disruption of mechanoreceptors and subsequent long-term alterations in neuromuscular control may contribute to these deficits, and if inadequately addressed, may increase the risk of post-traumatic osteoarthritis and progressive joint degeneration, adversely affecting quality of life. Consequently, FAI has emerged as an important and increasingly recognized public health concern.

Over the past few decades, the prevalence of overweight and obesity has increased substantially worldwide. Recent large-scale analyses indicate that in 2021 an estimated 2.11 billion adults (aged ≥25 years) were living with overweight or obesity globally, and pooled international data further show continuing upward trends in obesity through 2022 (Collaboration N C D R F, 2024; Collaborators G B D A B, 2025). Excess body weight has been identified as a risk factor for falls (Chang and Jen, 2022), which remain a leading cause of unintentional injury and death in daily and occupational settings. Biomechanically, higher body mass increases musculoskeletal loading and can compromise strength, balance, and mobility during walking and other activities of daily living. Overweight is also associated with abnormal joint-load distributions, particularly during weight-bearing tasks such as walking and stair negotiation (Khalaf et al., 2022).

The effects of excess weight on gait biomechanics have been examined extensively in otherwise healthy populations (Capodaglio et al., 2021; Singh et al., 2021). Evidence suggests that overweight and obesity are associated with adaptive gait changes, including alterations in stance-swing phase distribution and single- and double-support timing (Meng et al., 2017). In addition, obesity is a recognized risk factor for knee and hip osteoarthritis (Messier et al., 2022; Tsuchiya et al., 2023). Observational study further report that higher BMI is associated with an increased risk of sports-related injury (Messier et al., 2022; Bi et al., 2023). Given that individuals with FAI already exhibit impaired ankle stability and compromised functional support, excess body mass may plausibly further influence gait mechanics and compensatory strategies. However, gait-analysis findings in individuals with FAI remain inconsistent. Some studies report ankle-level changes during walking (e.g., altered subtalar inversion/eversion and reduced dorsiflexion with increased inversion) (Cao et al., 2019; Yin et al., 2024), whereas others find no differences in mean ankle angles, with alterations mainly reflected in greater kinematic variability during running (Hamacher et al., 2016). Under more demanding tasks (e.g., obstacle-crossing), individuals with FAI may show proximal compensations (e.g., greater trunk lateral flexion) and reduced mediolateral stability at landing (Ma et al., 2025). These inconsistencies may relate to differences in disability level, task conditions, and analytic methods, as also highlighted by recent systematic syntheses of ankle-instability gait biomechanics (Luan et al., 2024).

Because excess body mass increases lower-limb loading and can shift joint-load distributions during walking, it may exacerbate gait impairments in functional ankle instability. However, evidence specific to functional ankle instability remains limited and often focuses on distal ankle measures or isolated spatiotemporal parameters, without integrating global gait deviation/variability indices and proximal joint adaptations. Clarifying whether overweight is associated with a more proximal (pelvis/hip) compensatory strategy could inform more targeted rehabilitation and the potential role of weight management. Therefore, this study compared spatiotemporal parameters, gait deviation/variability indices, and sagittal-plane hip, knee, and ankle kinematics and kinetics during level walking between normal-weight and overweight adults with functional ankle instability. We hypothesized that overweight would be associated with greater pelvis/hip deviations and variability, with comparatively smaller between-group differences at the ankle.

## 2 Materials and methods

### 2.1 Study design

This study was a cross-sectional comparative analysis of baseline gait data collected within a registered clinical trial (ChiCTR2100041790). The protocol was approved by the Ethics Review Committee of Fujian Provincial Hospital (No. K2019-03-035). All participants provided written informed consent prior to participation.

### 2.2 participants

Participants were recruited from hospitals, community centers, and sports academies within the city. Purposive sampling was used to enhance participant diversity. Recruitment materials included online advertisements, posters displayed in hospitals and community settings, and community outreach initiatives.

### 2.3 Inclusion criteria

Participants were eligible if they met all of the following criteria: (1) at least one severe ankle sprain within the past year resulting in ≥1 day of activity limitation; (2) at least two episodes of “giving way” or perceived unilateral ankle instability within the past year; (3) Cumberland Ankle Instability Tool (CAIT) score ≤ 24; (4) no history of sprain or instability symptoms in the contralateral ankle; (5) the injured ankle was the right side, and the right side was the dominant limb; (6) no previous surgery and no evidence of mechanical instability on the injured side; and (7) age ≥ 18 years and willingness to participate.

### 2.4 Exclusion criteria

Participants were excluded if they met any of the following criteria: (1) history of lower-limb fracture or prior lower-limb surgery; (2) acute lower-limb injury or pain within the past two weeks; (3) known neuromuscular disease or vestibular dysfunction; or (4) mechanical ankle instability, defined as positive findings on both the anterior drawer test and the talar tilt test.

### 2.5 Outcome measurements

#### 2.5.1 Baseline collection

A total of 44 participants with FAI who met the inclusion and exclusion criteria were enrolled. Height was measured using a calibrated portable stadiometer (seca 213; SECA GmbH & Co. KG, Hamburg, Germany), and body mass was measured using a calibrated digital flat scale (seca 803; SECA GmbH & Co. KG, Hamburg, Germany) by a trained researcher; participants were barefoot and wore light clothing. Body mass index (BMI) was calculated as weight (kg) divided by height squared (m²). Participants were categorized as normal weight (BMI 18.5–24.0 kg/m²) or overweight (BMI 24.0–28.0 kg/m²) according to the Guidelines for Prevention and Control of Overweight and Obesity in Chinese Adults (Chen et al., 2004).

#### 2.5.2 Primary outcomes

##### 2.5.2.1 Pain intensity

Pain intensity was assessed using a 10-cm visual analogue scale (VAS) for pain, anchored at 0 (no pain) and 10 (worst imaginable pain). Participants indicated their current pain level on the scale, and the score was recorded as the distance (cm) from the 0 anchor.

##### 2.5.2.2 Functional status

Self-reported ankle instability was assessed using the CAIT, a validated questionnaire for evaluating perceived ankle instability. The CAIT yields a total score from 0 to 30, with lower scores indicating worse perceived stability; a score ≤24 is commonly used to identify FAI (Hiller et al., 2006).

Functional outcome was additionally evaluated using the American Orthopedic Foot & Ankle Society Ankle-Hindfoot Scale (AOFAS-AHS), which is widely used for conditions affecting the ankle and hindfoot. The AOFAS-AHS has demonstrated good reliability, with reported intraclass correlation coefficients ranging from 0.89 to 0.97 (Erichsen et al., 2020).

#### 2.5.3 Secondary outcomes

##### 2.5.3.1 Gait analysis

**Figure 1:**
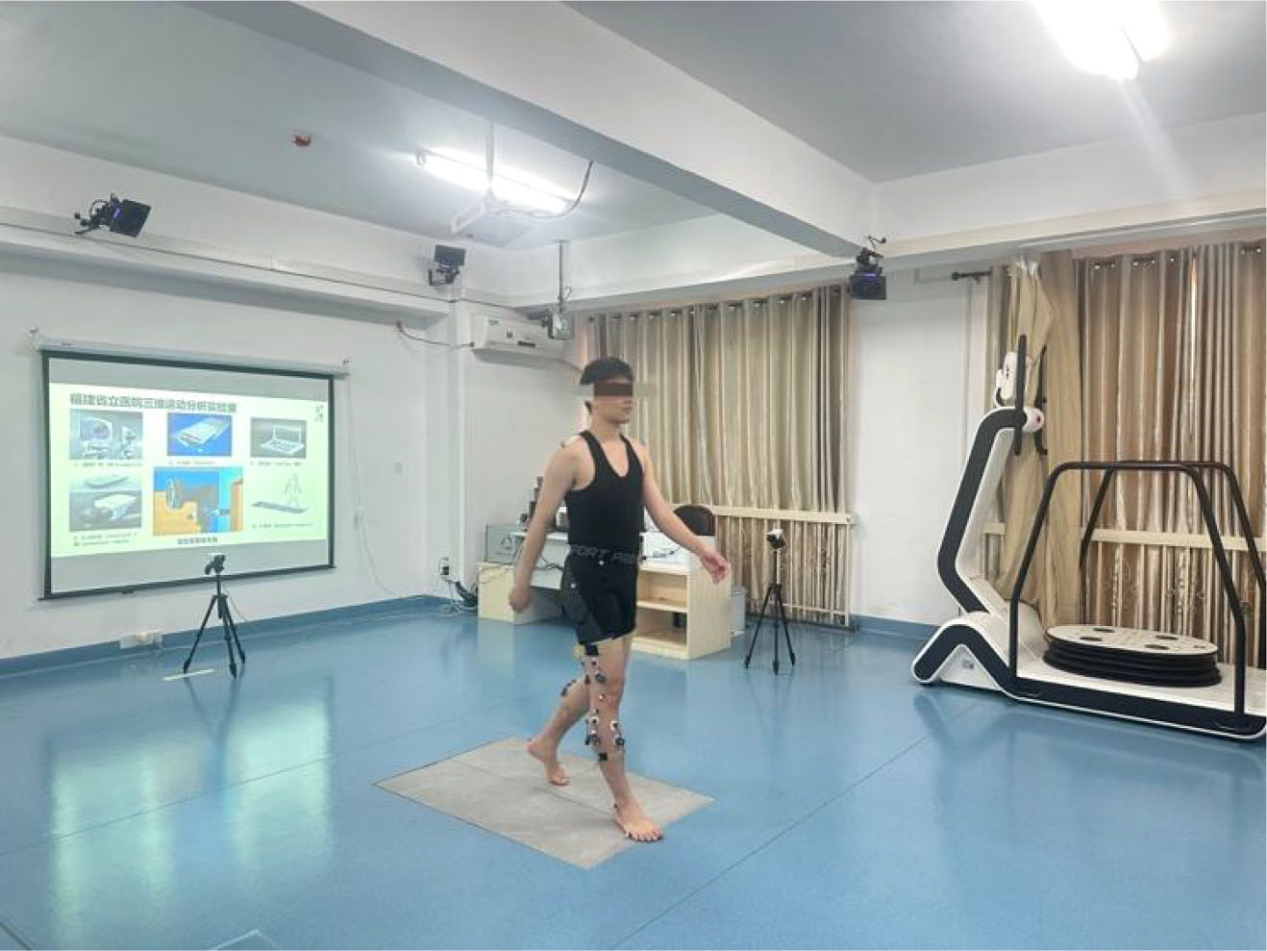
Marker set and gait acquisition process. Rigid three-marker clusters were secured on the lateral thigh and shank (partly covered by elastic wraps).

Gait data were collected using a SMART-D 400 infrared motion-capture system (BTS Bioengineering, Milan, Italy) synchronized with four force plates (BTS P6000D). A modified cluster-based (CAST-like) marker protocol was used: rigid three-marker clusters were secured on the lateral thigh and shank to reduce soft-tissue artifact, and anatomical landmarks were palpated and recorded during a static calibration. Marker placement followed a modified lower-limb model implemented in BTS SMART software; markers and clusters were fixed using hypoallergenic double-sided tape and elastic wraps (**Figure 1**). Because elastic wraps were used to secure clusters, clusters may be partially obscured in the acquisition photograph. Participants walked along an 8-m walkway at a self-selected comfortable speed. After an acclimation period, gait trials were recorded to obtain synchronized spatiotemporal and kinematic data. Spatiotemporal outcomes included stride time, stance time, swing time, walking speed, cadence, stride length, step length, and step width.

Marker trajectories were processed in BTS SMART software and visually inspected; short gaps were interpolated when feasible, whereas trials with excessive marker loss were excluded. Kinematic trajectories were low-pass filtered using a zero-lag, fourth-order Butterworth filter (6 Hz). Segment coordinate systems (pelvis, thigh, shank, and foot) were defined from the marker set during static calibration, and hip/knee/ankle joint angles were calculated using a Cardan XYZ sequence (X: flexion/extension; Y: abduction/adduction; Z: internal/external rotation). Kinematic and kinetic waveforms were time-normalized to 0–100% of the gait cycle, and discrete outcomes (e.g., peak angles/moments and toe-off angles) were extracted from the normalized curves. Sagittal-plane joint moments were computed by inverse dynamics and normalized to body mass (N·m/kg).

To characterize gait deviation and variability, we calculated the gait deviation index (GDI), which quantifies the overall deviation of pelvic and lower-limb kinematics from a reference dataset (Schwartz and Rozumalski, 2008). The gait profile score (GPS) was used to summarize overall kinematic deviation, and the gait variable score (GVS) was used to quantify deviations for specific kinematic components (e.g., pelvic tilt/rotation; hip adduction–abduction, flexion–extension, and rotation; knee flexion–extension; and ankle dorsiflexion–plantarflexion) (Baker et al., 2009). Sagittal-plane joint angles and ranges of motion for the hip, knee, and ankle were extracted. Kinetic outcomes included peak hip, knee, and ankle flexion/extension moments over the gait cycle, normalized to body mass (N·m/kg).

### 2.6 Statistical analysis

IBM SPSS Statistics 26.0 (IBM Corp., Armonk, NY, USA) was used for statistical analyses. Normality was assessed using the Shapiro-Wilk test. Between-group comparisons were performed using independent-samples t tests for normally distributed variables and Mann-Whitney U tests for non-normally distributed variables. For spatiotemporal parameters and gait biomechanics outcomes involving multiple tests, we controlled the family-wise error rate at 0.05 using the Holm-Bonferroni procedure within prespecified outcome families (spatiotemporal parameters; gait deviation/variability indices; and discrete sagittal-plane kinematics/kinetics). Accordingly, Holm–Bonferroni–adjusted p values (P_adj) were calculated and reported for all outcomes subjected to multiplicity control, and two-tailed significance was defined as P_adj < 0.05. For gait-cycle waveforms, we used SPM1D two-sample t tests with random field theory to control the family-wise error rate across the 0–100% gait cycle (two-tailed; interpolation enabled) (Pataky, 2010). Because three primary joint waveforms (hip, knee, and ankle sagittal-plane flexion/extension kinematics) were tested, the test-wise alpha was Bonferroni-adjusted to 0.0167 (0.05/3); SPM1D controlled family-wise error across time within each waveform at this threshold.

## 3 Results

### 3.1 Baseline characteristics

A total of 44 participants with FAI were included, with 22 in the normal-weight group and 22 in the overweight group. The normal-weight group comprised 9 females and 13 males, and the overweight group comprised 13 females and 9 males. There were no significant between-group differences in age (46.0 ± 20.13 vs 51.0 ± 20.14 years, respectively; *P* ≥ 0.05). As expected, BMI was significantly higher in the overweight group (21.27 ± 1.63 vs 26.08 ± 1.27 kg/m²; *P* < 0.05).

With respect to clinical outcomes, the overweight group demonstrated worse ankle function and symptoms, with significantly lower CAIT scores (17.86 ± 3.66 vs 14.59 ± 4.93; *P* < 0.05), higher VAS pain scores (2.14 ± 1.78 vs 3.55 ± 1.87; *P* < 0.05), and lower AOFAS-AHS scores (84.73 ± 6.68 vs 78.36 ± 11.00; *P* < 0.05). Baseline characteristics are summarized in **Table 1**.

**Table 1:** Baseline characteristics.

Characteristics	Normal weight (n=22)	Overweight (n=22)	z	*P*
Age(year)	46(20.13)	51(20.14)	0.78	0.44
Male/female	13/9	9/13	1.19	0.23
Injured side (right/Left)	22/0	22/0	0	1
BMI(kg/m^2^)	21.17(1.63)	26.08(1.27)	-5.68	**<0.01**
CAIT	17.86(3.66)	14.59(4.93)	-2.16	**0.03**
AOFAS	84.73(6.68)	78.36(11.00)	-2.16	**0.03**
VAS	2.14(1.78)	3.55(1.87)	2.52	**0.01**

The bold values indicate a statistically significant difference (P < 0.05).

### 3.2 Spatiotemporal parameters

As shown in **Table 2**, unadjusted comparisons suggested a small between-group difference in walking speed (P < 0.05). After Holm–Bonferroni correction within the spatiotemporal outcome family, none of the spatiotemporal parameters differed significantly between groups (all P_adj ≥ 0.05).

**Table 2:** Spatiotemporal parameters in normal weight and overweight participants with FAI (mean ± SD).

Parameter	Normal weight (n=22)	Overweight (n=22)	Z value	P/ P_adj
Stride Time (s)	1.12(0.09)	1.08(0.09)	-1.53	0.1264/ 0.7587
Stance Time (s)	0.68(0.07)	0.64(0.09)	-2.09	0.0364/ 0.2881
Swing Time (s),	0.43(0.03)	0.44(0.06)	0.26	0.7952/ 1.0000
Mean Velocity(m/s)	1.10(0.15)	0.98(0.17)	-2.10	0.0360/ 0.2881
Cadence(steps/min)	108.75(8.94)	111.48(8.77)	1.15	0.2499/ 1.0000
Stride Length (m)	1.15(0.15)	1.13(0.21)	-0.05	0.9625/ 1.0000
Step Length (m)	0.57(0.08)	0.57(0.10)	0.48	0.6299/ 1.0000
Step Width (m)	0.10(0.05)	0.09(0.03)	-0.07	0.9436/ 1.0000

The impacted side of FAI comprised exclusively the right legs.

*Statistically significant between-group difference after Holm–Bonferroni correction (P_adj < 0.05). P values are shown as P (unadjusted) and P_adj (Holm-adjusted) in the same column (top/bottom); Holm–Bonferroni was applied within this table.

### 3.3 Gait variability and deviation metrics

In **Table 3**, several deviation components showed nominal between-group differences (e.g., hip abduction–adduction and hip flexion–extension on the affected side), but none survived Holm–Bonferroni correction across deviation/variability indices (all P_adj ≥ 0.05).

**Table 3:** Gait deviation and variability indices in normal-weight and overweight participants with FAI (mean ± SD).

Variable	Normal weight (n=22)		Overweight (n=22)		Right side between groups		Left side between groups	
	Right	Left	Right	Left	Z value	P/ P_adj	Z value	P/ P_adj
Gait Deviation Index,mean(SD)	89.71(8.88)	89.63(11.38)	89.14(12.33)	87.52(18.67)	0.73	0.4668/ 1.0000	0.56	0.5732/ 1.0000
Gait Profile Score (deg),mean(SD)	8.11(1.93)	8.38(2.49)	8.2(3.17)	8.41(2.63)	-2.21	0.0272/ 0.2451	0.07	0.9438/ 1.0000
Pelvis Obliquity (deg),mean(SD)	1.95(0.64)	3.2(1.31)	2.15(1.02)	2.45(0.97)	1.87	0.0617/ 0.4936	-0.07	0.9438/ 1.0000
Pelvis Tilt (deg),mean(SD)	7.20(5.46)	6.69(4.30)	7.18(5.47)	6.19(4.19)	-0.63	0.5260/ 1.0000	-0.74	0.4595/ 1.0000
Pelvis Rotation (deg),mean(SD)	3.96(1.13)	4.18(1.34)	4.1(1.08)	4.35(1.42)	-0.28	0.7780/ 1.0000	-0.35	0.7245/ 1.0000
Hip Ab-Adduction (deg),mean(SD)	3.92(2.02)	6.53(2.87)	5.5(2.83)	5.15(2.09)	2.40	0.0166/ 0.1825	1.02	0.3071/ 1.0000
Hip Flex-Extension (deg),mean(SD)	8.55(4.69)	6.10(2.73)	8.58(6.06)	9.31(6.13)	-2.36	0.0183/ 0.1827	0.06	0.9532/ 1.0000
Hip Rotation (deg),mean(SD)	10.56(5.97)	11.62(5.72)	11.48(8.94)	10.04(4.08)	-0.40	0.6897/ 1.0000	0.25	0.8053/ 1.0000
Knee Flex-Extension (deg),mean(SD)	8.37(2.95)	8.89(4.75)	9.13(4.36)	10.24(5.42)	-0.81	0.4179/ 1.0000	0.08	0.9345/ 1.0000
Ankle Dorsi-Plantarflex (deg),mean(SD)	7.89(4.07)	7.33(3.19)	6.68(2.41)	6.76(3.23)	-1.02	0.3069/ 1.0000	-0.63	0.5260/ 1.0000
Foot Progression (deg),mean(SD)	8.16(4.38)	8.32(3.78)	7.21(4.22)	9.05(4.54)	-0.52	0.6054/ 1.0000	1.23	0.2175/ 1.0000

The term “right” refers to the 22 affected normal weight FAI legs and the 22 affected overweight FAI legs. Similarly, the term “left” refers to the 22 healthy side legs of a normal-weight FAI versus the 22 healthy, overweight legs of a FAI.

P values are shown as P (unadjusted) and P_adj (Holm-adjusted) in the same column (top/bottom). Holm–Bonferroni was applied within each side (m = 11 indices per side), with significance defined as P_adj < 0.05 (two-tailed).

### 3.4 Sagittal-plane kinematics and kinetics of the hip, knee, and ankle

Kinematic and kinetic results are summarized in **Table 4**. Unadjusted comparisons indicated nominal between-group differences for selected discrete sagittal-plane variables. After Holm–Bonferroni correction across discrete sagittal-plane variables, the overweight group demonstrated lower peak hip flexion (P_adj < 0.05). Hip flexion angle at toe-off showed a nominal between-group difference, but it did not remain significant after correction (P_adj ≥ 0.05). Discrete knee and ankle kinematics/kinetics did not differ significantly between groups after correction (all P_adj ≥ 0.05).

**Table 4:** Sagittal-plane kinematics and kinetics of the hip, knee, and ankle during walking in participants with FAI (mean ± SD).

Variable	Normal weight (n=22)	Overweight (n=22)	z	P/ P_adj
Hip kinematics and kinetics in the sagittal plane, degree.				
Hip peak flexion angle (degree)	43.98(4.71)	37.02(7.06)	3.2046	0.0014/ 0.0243
Hip peak extension angle (degree)	2.42(10.69)	-2.8(9.57)	1.3265	0.1847/ 1.0000
Hip ROM (degree)	41.55(9.99)	39.82(8.26)	1.0563	0.2908/ 1.0000
Hip peak flex-extension moment (N.m/kg)	0.75(0.18)	0.81(0.22)	-0.8791	0.3793/ 1.0000
Hip toe-off angle (degree)	41.6(5.42)	35.75(7.06)	2.7588	0.0058/ 0.0986
Hip heel strike angle (degree)	6.43(10.41)	1.05(10.09)	1.3146	0.1887/ 1.0000
Knee kinematics and kinetics in the sagittal plane, degree.				
Knee peak flexion angle (degree)	62.54(6.96)	58.15(9.33)	1.8898	0.0588/ 0.9405
Knee minimum extension angle(degree)	5.55(7.99)	3.63(8.72)	0.5165	0.6055/ 1.0000
Knee ROM (degree)	58.5(9.16)	54.52(10.01)	1.7606	0.0783/ 1.0000
Knee peak flex-extension moment (N.m/kg)	0.7(0.31)	0.65(0.49)	0.7906	0.4292/ 1.0000
Knee toe-off angle (degree)	9.81(8.38)	7.92(7.95)	0.7512	0.4525/ 1.0000
Knee heel strike angle (degree)	32.4(17.15)	24.14(11.35)	1.3850	0.1661/ 1.0000
Ankle kinematics and kinetics in the sagittal plane, degree.				
Ankle peak dorsiflexion (degree)	16.40(6.31)	15.46(5.71)	0.2348	0.8144/ 1.0000
Ankle peak plantar flexion (degree)	-15.03(7.80)	-13.96(9.38)	-0.3756	0.7072/ 1.0000
Ankle ROM (degree)	31.43 (5.81)	29.42(9.62)	0.5751	0.5652/ 1.0000
Ankle peak dors-dorsiflexion moment (N.m/kg)	1.56(0.37)	1.53(0.27)	-0.0355	0.9717/ 1.0000
Ankle toe-off angle (degree)	-7.05(8.95)	-3.27(10.39)	-1.1620	0.2452/ 1.0000
Ankle heel strike angle (degree)	1.17(4.81)	0.62(5.22)	-0.9978	0.3184/ 1.0000

P values are reported as P (unadjusted)/P_adj (Holm-adjusted) in the same column (top/bottom). Holm–Bonferroni adjustment was applied within the prespecified **Table 4** outcome family (discrete sagittal-plane kinematic and kinetic variables), with significance defined as P_adj < 0.05 (two-tailed).

### 3.5 Trajectory-level gait-cycle kinematics and kinetics

**Figure 2:**
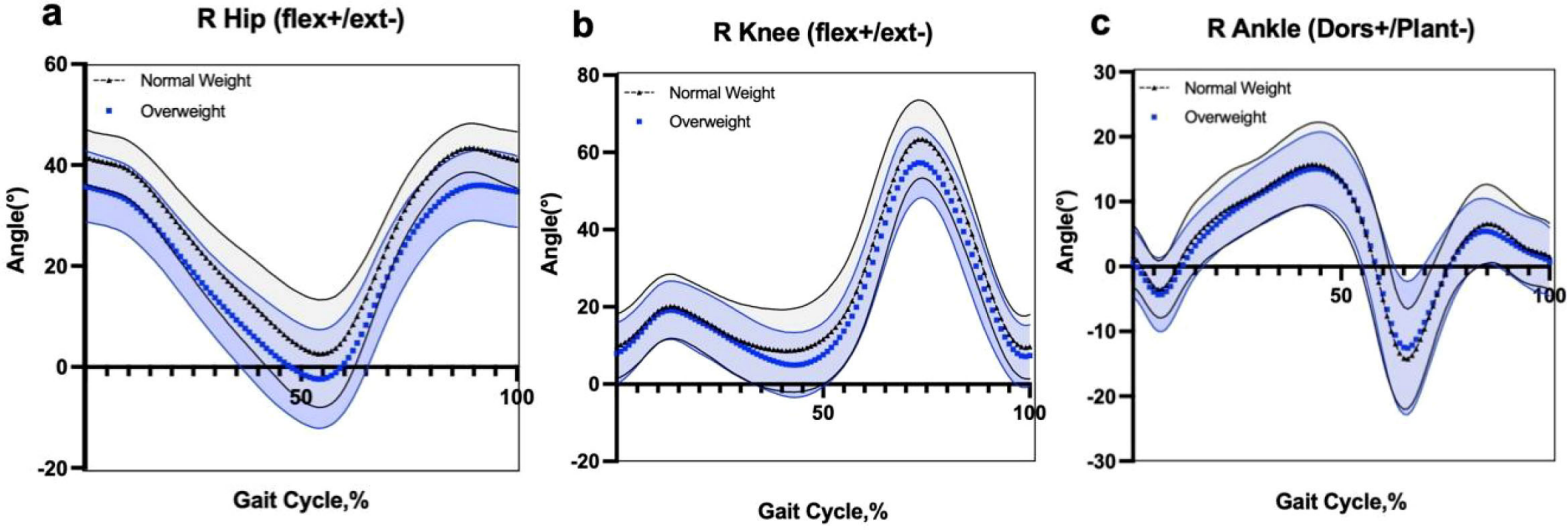
Sagittal-plane lower-limb kinematics of the affected limb during walking in normal-weight and overweight participants with FAI. **(a–c)** depict the sagittal-plane kinematics of the hip, knee, and ankle, respectively. Group mean waveforms are shown as lines, with shaded bands indicating ±1 SD. Waveform-level inference was performed using SPM1D (random field theory; two-tailed; Bonferroni-adjusted α = 0.0167). No significant clusters were detected across the gait cycle at this threshold; therefore, no background shading is used to denote statistical significance. dors, dorsiflexion; plant, plantarflexion; ext, extension; flex, flexion.

Trajectory-level sagittal-plane hip, knee, and ankle kinematics of the affected limb across the normalized gait cycle are shown in **Figure 2**. Waveform-level between-group inference was performed using SPM1D two-sample t tests with random field theory (two-tailed; interpolation enabled). To account for three joint waveforms (hip, knee, and ankle flexion/extension kinematics), the test-wise alpha was set to 0.0167 (0.05/3). No supra-threshold clusters were detected for hip, knee, or ankle kinematics across the gait cycle at this threshold (all set-level p = 1.000).

## 4 Discussion

The primary aim of this study was to characterize gait biomechanics in overweight individuals with FAI, with particular emphasis on how excess body mass influences lower-limb joint behavior during walking. Using synchronized three-dimensional motion capture and force-plate measurements, we quantified spatiotemporal parameters, kinematics, kinetics, and gait-variability metrics, and performed waveform-level SPM1D analyses of sagittal-plane hip, knee, and ankle flexion/extension angle trajectories between normal-weight and overweight participants with FAI. Focusing on the sagittal plane facilitated a clear interpretation of joint motion patterns aligned with the direction of progression during gait.

### 4.1 Overweight increases mechanical demands and is associated with mobility-related gait adaptations in FAI

Excess body mass increases the mechanical work required for locomotion and is often accompanied by higher absolute ground-reaction forces and greater lower-limb joint moments during walking (Bode et al., 2020). In adults and children, overweight/obesity has been associated with a slower self-selected walking speed and longer stance and/or double-support durations, together with shorter steps/strides and a wider base of support (Dufek et al., 2012; Kim et al., 2022). These temporospatial adjustments are commonly interpreted as stability-oriented and load-management strategies (Stamenkovic et al., 2021). While such adaptations may help regulate balance and reduce joint stress at a given task demand, they can also lower walking efficiency and overall ambulatory capacity. For individuals with functional ankle instability, who already demonstrate altered lower-limb biomechanics and impaired neuromuscular control during gait, added body mass may further challenge regulation of the center of mass over the base of support (Lugade et al., 2011).

In the present study, unadjusted analyses suggested a nominal reduction in mean walking speed in the overweight group, whereas the remaining spatiotemporal parameters differed only minimally. After controlling the family-wise error rate within the spatiotemporal outcome family using the Holm-Bonferroni procedure, none of the spatiotemporal parameters remained statistically significant. This pattern suggests that BMI-related differences in spatiotemporal gait during comfortable level walking, if present, are modest in magnitude in individuals with functional ankle instability. Several factors may contribute, including a predominance of overweight rather than severe obesity, compensatory behavior at self-selected walking speed, and reduced statistical power after multiplicity control for small effects. It is also plausible that overweight-related mobility adaptations in ankle instability become more evident under higher-demand conditions (e.g., fast walking, uneven surfaces, obstacle negotiation, fatigue, or dual-tasking), where balance control is stressed and lower-limb loading increases. Future studies should therefore recruit a wider BMI spectrum and incorporate ecologically challenging tasks to clarify when and how excess body mass amplifies gait adaptations in functional ankle instability.

### 4.2 Excess body mass alters gait-cycle joint kinematics in FAI, with prominent proximal effects

A key finding was that overweight participants with FAI exhibited reduced hip flexion-related measures. In discrete outcomes, peak hip flexion remained significant after Holm–Bonferroni correction, whereas the hip flexion angle at toe-off showed a nominal between-group difference but did not remain significant after correction. At the waveform level, SPM1D did not identify any supra-threshold clusters for hip, knee, or ankle sagittal-plane kinematics across the gait cycle at the Bonferroni-adjusted alpha (0.0167), suggesting that between-group differences were not sustained across the time-series domain under confirmatory waveform inference.

These findings support the notion that excess body mass may be associated with a more proximal (hip-level) strategy during level walking in FAI. Initial contact and the transition from stance to swing are phases in which dynamic stability is challenged, and instability-related perturbations have been linked to increased fall risk (Nagano et al., 2013). Because postural control is impaired in FAI and overweight is associated with greater postural sway, added mass may increase the demand to regulate the center of mass over the base of support (Kim et al., 2025). Our data suggest that this additional demand is expressed primarily as reduced hip flexion/excursion rather than consistent distal (ankle) alterations, broadly consistent with prior observations that overweight can preferentially influence proximal mechanics in clinical populations and that higher body weight can modify hip loading/kinematics across the gait cycle (Eckel et al., 2012).

Reduced hip excursion may represent a stability-oriented adaptation (e.g., increased limb stiffness or altered motor control) but could also compromise toe clearance and limit functional reserve during more demanding walking conditions. Notably, reduced hip flexion has also been reported in overweight/obese individuals compared with normal-weight controls (Villarrasa-Sapina et al., 2016). From a rehabilitation perspective, these results support a focus on proximal strengthening and neuromuscular-control training in overweight individuals with FAI, alongside conventional ankle-focused rehabilitation. Integrative neuromuscular programs in youth with overweight/obesity have been shown to improve gait biomechanics (Molina-Garcia et al., 2022), and exercise interventions targeting lower-extremity function can modify gait-related outcomes in pediatric obesity (Horsak et al., 2019).

Although FAI has been associated with limited ankle dorsiflexion compared with healthy controls (Ziaei Ziabari et al., 2022), our discrete outcomes did not indicate that overweight further diminished ankle dorsiflexion or sagittal-plane ankle kinetics in this cohort after correction. In exploratory trajectory-level analyses, the overweight group showed lower contralateral knee flexion during swing and a tendency toward reduced contralateral ankle dorsiflexion during mid-stance, which may reflect compensatory strategies to maintain whole-body stability and manage load when the affected side provides insufficient support. Similar reductions in knee excursion have been reported in overweight populations during walking (Capodaglio et al., 2021; Tramonti et al., 2022; Adouni et al., 2024), suggesting that contralateral adaptations may emerge even when peak-metric differences are not robust after multiplicity control.

### 4.3 Joint kinetics and gait deviation/variability indices in overweight individuals with FAI

After Holm–Bonferroni correction across gait deviation/variability indices, no indices differed significantly between groups. Several components showed nominal (unadjusted) between-group differences (e.g., hip-related GVS components on the affected side), but these did not remain significant after multiplicity control, suggesting that any BMI-related effects on these global deviation/variability summaries are small during comfortable level walking.

For joint kinetics, we did not observe significant between-group differences in peak sagittal-plane hip, knee, or ankle moments after correction. This aligns with reports that obesity is not necessarily associated with higher knee joint torque/power during level walking (Capodaglio et al., 2021) and that mass-normalized internal mechanical work can be preserved even in severe obesity (Fernandez Menendez et al., 2020). At the same time, studies in healthy overweight/obese cohorts have reported higher plantar loading and peak pressures with increasing BMI (Khalaf et al., 2022), indicating that excess mass can increase mechanical demands even when normalized peak-moment metrics appear similar.

One possible interpretation of our results is that in overweight individuals with FAI, compensatory strategies (e.g., a tendency toward slower walking speed and constrained joint excursions) may help maintain peak joint moments within a similar range despite increased body mass. In addition, the added mechanical load associated with excess weight may alter movement patterns in ways that reduce the sensitivity of peak-moment measures to underlying sensorimotor deficits. Notably, our cohort comprised overweight (rather than obese) participants, and the mass difference between groups may have been insufficient to elicit detectable changes in peak joint moments during level walking. Future studies including individuals with obesity and more demanding locomotor tasks (e.g., fast walking, uneven terrain, or prolonged walking) may be better positioned to determine whether excess mass amplifies kinetic impairments and gait deviations in FAI.

## 5 Limitations

Several limitations should be acknowledged. First, the sample size was modest, potentially limiting power to detect small BMI-related effects; larger, adequately powered cohorts are needed to confirm and generalize these findings. Second, surface electromyography was not collected, so muscle activation timing and co-contraction mechanisms underlying the kinematic adaptations could not be examined. Third, gait was evaluated only during level walking at a self-selected comfortable speed, which may underestimate adaptations that appear under higher task demands; future studies should test multiple speeds and challenging contexts (e.g., fast walking, uneven terrain, prolonged walking, dual-tasking). Fourth, participants were overweight only; inclusion of individuals with obesity would allow assessment of dose-response relationships. Finally, although we applied Holm–Bonferroni adjustment for discrete outcomes and used SPM1D (random field theory) for waveform-level inference, waveform analyses were limited to sagittal-plane flexion/extension kinematics and to three prespecified joint waveforms; future studies could extend confirmatory waveform inference to additional planes and kinetic waveforms.

## 6 Conclusion

In summary, after controlling for multiple testing within prespecified outcome families, spatiotemporal gait parameters and gait deviation/variability indices were largely comparable between normal-weight and overweight individuals with FAI. Among discrete sagittal-plane outcomes, the most robust between-group difference was observed at the hip, with overweight participants demonstrating lower peak hip flexion (P_adj < 0.05), while other outcomes showed no significant between-group differences after correction. These findings suggest that modest excess body mass may be associated primarily with proximal hip kinematic adaptations in FAI, which may be relevant when interpreting gait biomechanics and designing prevention and rehabilitation programs.

## Data Availability

The raw data supporting the conclusions of this article will be made available by the authors, without undue reservation.
